# Glymphatic dysfunction across sleep disorders: a meta-analysis of DTI-ALPS studies

**DOI:** 10.3389/fneur.2026.1789842

**Published:** 2026-04-15

**Authors:** Xiaoxin Zhang, Dongmei He

**Affiliations:** 1 Department of Radiology, The Second Clinical Medical College, North Sichuan Medical College, Nanchong, China; 2 Department of Neurology, The Second Clinical Medical College, North Sichuan Medical College, Nanchong, China

**Keywords:** diffusion tensor imaging analysis along the perivascular space, DTI-ALPS, glymphatic dysfunction, meta-analysis, sleep disorders

## Abstract

**Background:**

Sleep disorders are increasingly linked to glymphatic dysfunction, but whether this impairment is universal across different sleep pathologies remains unclear.

**Objective:**

This meta-analysis examined whether glymphatic dysfunction, assessed via the diffusion tensor imaging analysis along the perivascular space (DTI-ALPS) index, may represent a shared neural feature across a spectrum of sleep disorders.

**Methods:**

We systematically searched PubMed, EMBASE, and the Cochrane Library from inception to December 31, 2025, for observational studies comparing DTI-ALPS index between patients with sleep disorders and healthy controls. Data were synthesized from 19 studies (n = 2,315) using a random-effects model to calculate standardized mean differences (SMDs). Methodological quality was assessed with the Newcastle-Ottawa Scale. Subgroup analyses, meta-regression, sensitivity analyses, and publication bias assessment were performed.

**Results:**

The pooled analysis revealed significant global glymphatic impairment in patients with sleep disorders (SMD = −1.60, 95% CI [−2.65, −0.54], *p* = 0.003), indicating global glymphatic dysfunction. However, heterogeneity was extremely high (I^2^ = 94.7%). Disorder-specific analyses showed pronounced deficits in obstructive sleep apnea (SMD = −0.92, *p* < 0.001) and idiopathic REM sleep behavior disorder (SMD = −0.63, *p* = 0.004), as well as in PSQI-defined poor sleep (SMD = −0.50, *p* < 0.001). Results for insomnia and narcolepsy were non-significant and highly heterogeneous. Meta-regression identified no significant moderators; publication bias was detected (*p* = 0.01).

**Conclusion:**

Glymphatic dysfunction, as assessed by DTI-ALPS, is consistently observed across several sleep disorders, suggesting a potential shared pathway. However, extreme heterogeneity limits interpretability of the pooled effect, and cross-sectional data preclude causal inference.

## Introduction

Sleep disorders, as defined by the International Classification of Sleep Disorders (ICSD-3), extend beyond mere disruptions of normal sleep patterns; they represent pervasive challenges to central nervous system homeostasis with profound implications for long-term brain health (1). Beyond the well-established consequences of sleep fragmentation, an emerging pathophysiological model positions dysregulation of the brain’s glymphatic system as a central mechanism linking disordered sleep to neurodegenerative risk (2, 3).

The glymphatic system is a macroscopic waste-clearance network that facilitates efficient exchange of cerebrospinal fluid (CSF) and interstitial fluid (ISF) through specialized perivascular tunnels (4). Its activity is strongly sleep-state dependent, with solutes, including neurotoxic metabolites such as amyloid-*β*, being cleared most effectively during slow-wave sleep (3). Consequently, chronic sleep disruption is hypothesized to induce a state of functional glymphatic insufficiency, creating a permissive environment for pathological protein accumulation and increasing susceptibility to a range of neurological disorders (5, 6). Within this framework, measurable alterations in glymphatic function may serve as a candidate biomarker; however, cross-sectional associations do not establish diagnostic validity, and longitudinal data are lacking.

The recent introduction of Diffusion Tensor Imaging Analysis along the Perivascular Space (DTI-ALPS) has provided a transformative, non-invasive method to assess human glymphatic dynamics *in vivo*. By quantifying water-diffusion anisotropy specifically along the perivascular spaces of deep medullary veins, the DTI-ALPS index acts as a proxy for glymphatic fluid-transport efficiency (7). Its validation in neurodegenerative contexts renders it an ideal tool for investigating the sleep–glymphatic–neurodegeneration axis.

Although the foundational link between sleep and glymphatic clearance is established, a critical question remains: Is glymphatic impairment a universal or disorder-specific feature across the spectrum of sleep pathologies? Current evidence remains fragmented, lacking a synthesized quantitative assessment. This meta-analysis therefore aims to definitively quantify and compare glymphatic activity, as measured by the DTI-ALPS index, across various sleep-disordered populations and healthy controls, and to elucidate whether glymphatic dysfunction constitutes a shared pathophysiological pathway.

## Materials and methods

Ethical approval was not required for this study as it was a meta-analysis based on data extracted from previously published studies, which did not involve direct contact with human participants or animals. This meta-analysis was conducted in accordance with the Preferred Reporting Items for Systematic Reviews and Meta-Analyses (PRISMA) guidelines (8).

### Literature search and eligibility criteria

A systematic literature search was performed across PubMed, EMBASE, and the Cochrane Library from inception until December 31, 2025. The search strategy employed a comprehensive combination of keywords on “DTI-ALPS” OR “diffusion tensor imaging analysis along the perivascular space” (Supplementary Table). The full search strings for each database are provided in the Supplementary materials.

Studies were included if they met the following pre-specified criteria: (1) enrolled adult human participants with a formal diagnosis of a sleep disorder according to ICSD-3 criteria, including insomnia, sleep-disordered breathing, hypersomnolence, circadian rhythm disorders, parasomnias, and sleep-related movement disorders; (2) Presented a cross-sectional comparison of glymphatic function between patient cohorts and healthy controls using the DTI-ALPS index; (3) provided extractable DTI-ALPS index data in the form of mean and standard deviation. No restrictions were placed on publication language or date. Studies enrolling participants with primary neurodegenerative diseases (Parkinson’s disease, Alzheimer’s disease) were included only if they reported a sleep disorder diagnosis as a secondary or comorbid condition; these were categorized as ‘mixed sleep disorders’ and subjected to sensitivity analysis.

### Data extraction and outcomes

Two independent reviewers (X. Z. and D. H.) extracted data from each included study using a standardized data extraction form. Discrepancies were resolved through discussion or, if necessary, consultation with a third reviewer. The following information was extracted: first author, year of publication, country, study design, sample size (patients and controls), demographic characteristics (age, sex distribution), sleep disorder diagnosis (as per ICSD-3 criteria), MRI field strength, DTI-ALPS index values (mean and standard deviation for each group), and methodological quality indicators (Newcastle–Ottawa Scale scores, NOS). The NOS evaluates studies across three key domains: sample selection (0–4 stars), comparability of groups (0–2 stars), and ascertainment of the exposure or outcome (0–3 stars), with a higher score indicating superior quality (9).

The primary outcome was the glymphatic function, quantified by the DTI-ALPS index. The DTI-ALPS index is a continuous measure derived from diffusion tensor imaging, reflecting water diffusivity along perivascular spaces; lower values indicate greater glymphatic impairment. For each study, we extracted the mean DTI-ALPS index and its standard deviation for both the sleep disorder group and the healthy control group.

### Statistical analysis

All analyses were performed using R software (version 4.2.0) with the “meta” package. The primary effect measure was the standardized mean difference (SMD) in the DTI-ALPS index between patients with sleep disorders and healthy controls. A random-effects model was employed to account for anticipated clinical and methodological heterogeneity. Statistical heterogeneity was quantified using the I^2^ statistic.

To assess the potential impact of publication bias, we performed both Egger’s regression test and trim-and-fill analysis. Pre-specified meta-regression analyses were conducted to explore potential sources of heterogeneity. The robustness of the pooled findings was tested through sensitivity analyses, including leave-one-out procedures and subgroup analyses stratified by specific sleep-disorder diagnoses.

## Results

### Study selection and cohort characteristics

**Figure 1:**
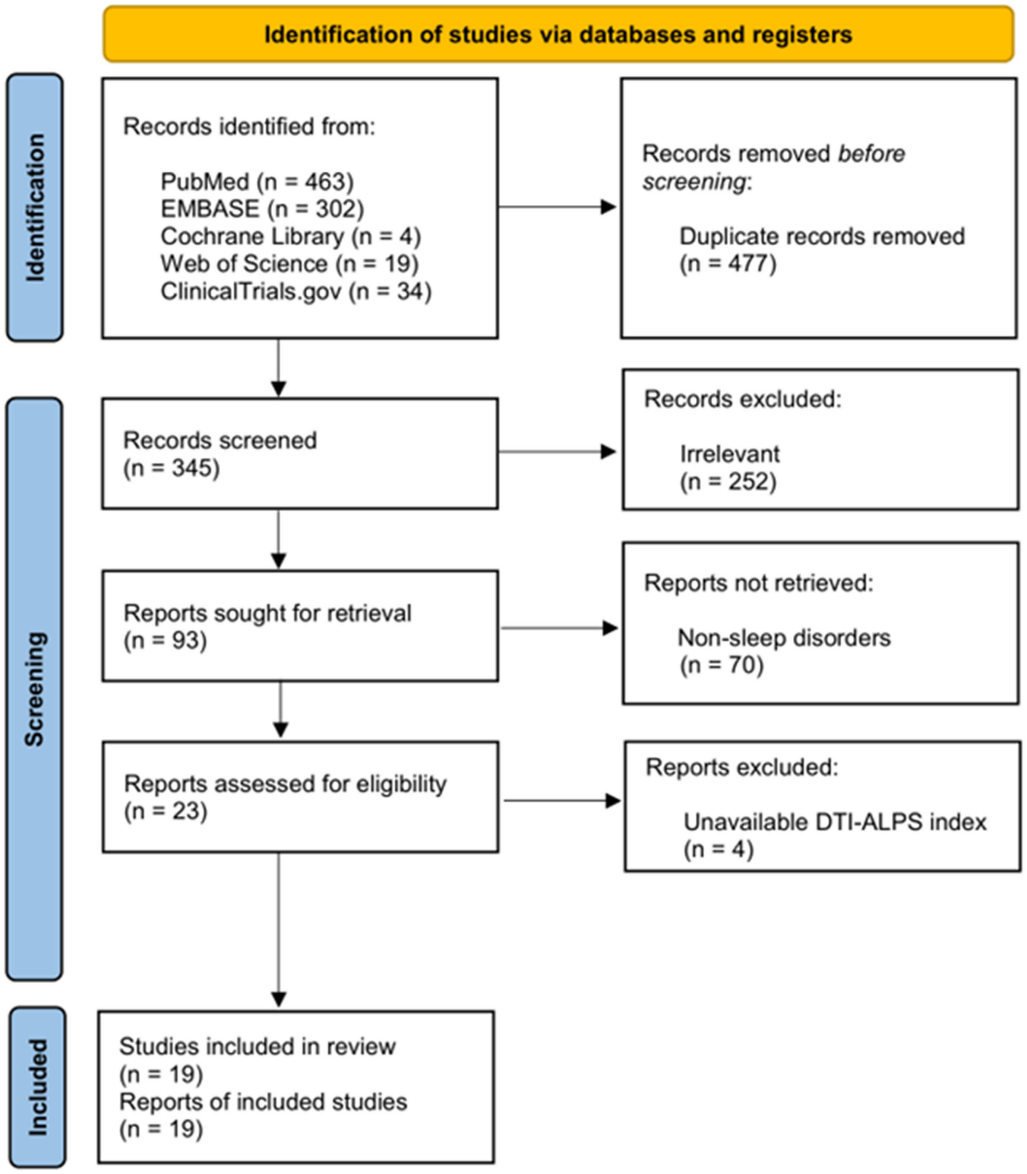
PRISMA flow diagram for the systematic review.

This systematic search identified 19 eligible studies (10–28), comprising a total of 2,315 participants (1,090 patients with sleep disorders and 1,225 healthy controls) (Figure 1). Included studies covered major sleep-disorder categories: four studies each for insomnia disorder and obstructive sleep apnea (OSA); three studies each for narcolepsy and idiopathic rapid eye movement sleep behavior disorder (iRBD); one for restless legs syndrome (RLS); two for mixed sleep disorders; and two that defined poor sleep quality based on a Pittsburgh Sleep Quality Index (PSQI) score >5 (Table 1; Supplementary Table).

**Table 1:** Characteristics of included studies.

Study	Sample size (Disorder/Control)	Age (years) Disorder/Control	Female (%) Disorder/Control	Spectrum	Quality
Li et al. (2024) (10)	114 (43/71)	62.7 ± 9.8/60.0 ± 9.3	25.6/43.7	Sleep disorder (mixed)	3/2/2
Shang et al. (2024) (11)	65 (40/25)	68.6 ± 6.2/67.4 ± 7.1	65.0/52.0	Sleep disorder (mixed)	3/2/2
Zhou et al. (2024) (12)	61 (29/32)	54.5 ± 1.5/55.5 ± 1.1	41.4/53.1	Insomnia disorder	4/1/2
Qian et al. (2024) (13)	49 (28/21)	71.7 ± 7.5/71.7 ± 7.5	57.1/57.1	Insomnia disorder	2/1/2
Yu et al. (2024) (14)	53 (33/20)	57.3 ± 5.3/58.0 ± 5.8	90.9/80.0	Insomnia disorder	4/2/2
Ruifang et al. (2025) (15)	62 (25/37)	52.9 ± 1.9/32.5 ± 2.7	68.0/59.5	Insomnia disorder	4/2/2
Ho-Joon et al. (2022) (16)	48 (24/24)	62.0 ± 15.0/63.7 ± 6.3	37.5/41.7	OSA	4/2/2
Bhaswati et al. (2022) (17)	121 (59/62)	49.9 ± 10.0/50.1 ± 10.4	40.7/45.2	OSA	4/2/2
Shiwei et al. (2024) (18)	155 (105/50)	33.4 ± 8.9/37.0 ± 8.6	23.8/34.0	OSA	4/2/2
Zhenliang et al. (2024) (19)	65 (31/34)	39.9 ± 9.3/43.2 ± 8.5	9.7/14.7	OSA	4/2/2
Ekim et al. (2022) (20)	36 (25/11)	33.5 ± 4.2/36.0 ± 6.8	60.0/54.5	Narcolepsy	4/2/2
Pengxin et al. (2024) (21)	83 (41/42)	19.0 ± 2.0/21.5 ± 1.5	46.3/57.1	Narcolepsy	4/2/2
Eva et al. (2025) (22)	23 (12/11)	33.3 ± 10.5/31.8 ± 13.4	33.3/36.4	Narcolepsy	4/2/2
Xiaoli et al. (2022) (23)	248 (119/129)	61.9 ± 7.6/62.0 ± 7.2	47.9/54.3	iRBD	3/2/2
Dong et al. (2022) (24)	36 (18/18)	69.3 ± 7.2/67.3 ± 6.2	38.9/38.9	iRBD	4/2/2
Yun Jung et al. (2023) (25)	40 (20/20)	73.0 ± 7.0/73.0 ± 6.9	40.0/40.0	iRBD	4/2/2
Kang Min (2023) (26)	120 (69/51)	57.0 ± 6.7/55.9 ± 8.1	71.0/74.5	RLS	4/2/2
Yuya et al. (2023) (27)	832 (317/515)	28.7 ± 3.5/28.8 ± 3.7	44.8/45.2	Poor sleep (PSQI>5)	3/2/2
Junko et al. (2024) (28)	104 (52/52)	73.1 ± 5.7/73.8 ± 4.3	57.7/57.7	Poor sleep (PSQI>5)	3/2/2

OSA, obstructive sleep apnea. iRBD, idiopathic rapid eye movement sleep behavior disorder. PSQI, Pittsburgh sleep quality index. RLS, restless legs syndrome.

### Methodological quality and heterogeneity profile

Methodological quality, assessed via the Newcastle–Ottawa Scale (NOS), was consistently high (scores ranging from 5 to 8 stars; mean ± SD: 7.5 ± 0.8), indicating robust study designs with rigorous participant selection, appropriate adjustment for comparability, and valid ascertainment of the DTI-ALPS outcome.

### Global and disorder-specific glymphatic impairment

**Figure 2:**
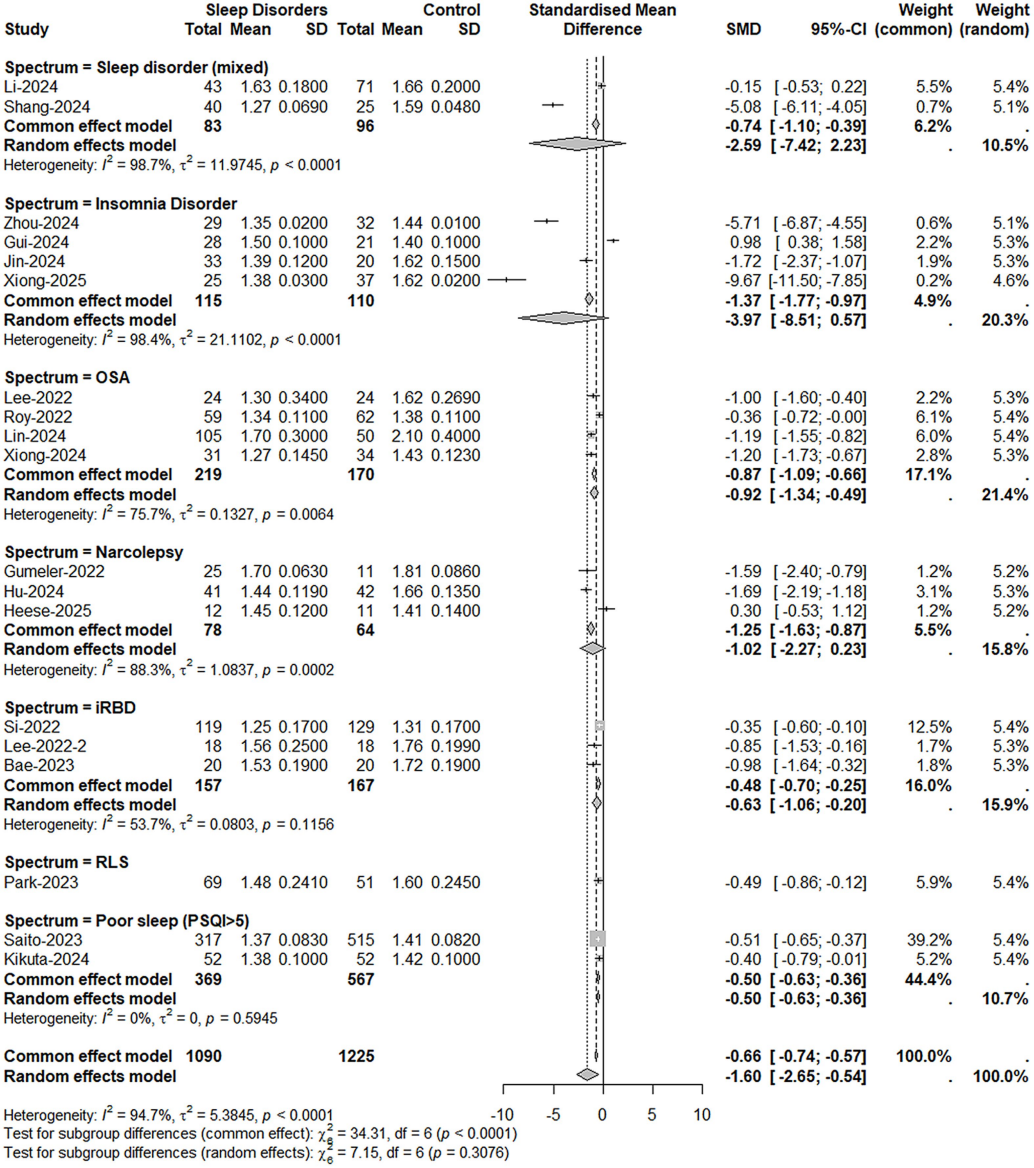
Forest plot of the pooled DTI-ALPS index using standardized mean difference (SMD) and 95% confidence interval (CI). The pooled analysis demonstrated significant DTI-ALPS reductions in overall sleep disorders (SMD = −1.60, 95% CI [−2.65, −0.54], *p* = 0.003; *I*^2^ = 94.7%), with pronounced deficits in OSA (*p* < 0.001), iRBD (*p* = 0.004), and PSQI-defined poor sleep (*p* < 0.001). Insomnia disorder (*p* = 0.086) and narcolepsy (*p* = 0.111) showed non-significant trends.

Primary meta-analysis revealed significant global impairment of glymphatic function in patients with sleep disorders compared to healthy controls, evidenced by a marked reduction in the pooled DTI-ALPS index (SMD = −1.60, 95% CI [−2.65, −0.54], *p* = 0.003). The extreme heterogeneity observed (I^2^ = 94.7%) likely reflects underlying pathophysiological diversity across sleep disorders rather than statistical artifact (Figure 2).

Subgroup analyses indicated a gradient of glymphatic compromise across specific diagnoses. The most pronounced deficits were observed in OSA (SMD = −0.92, *p* < 0.001; I^2^ = 75.7%) and iRBD (SMD = −0.63, *p* = 0.004; I^2^ = 53.7%)—disorders with strong mechanistic links to intermittent hypoxia and protein aggregation, respectively. PSQI-defined poor sleep also showed significant, albeit more modest, impairment (SMD = −0.50, *p* < 0.001; I^2^ = 0%).

In contrast, pooled estimates for insomnia disorder (SMD = −3.97, *p* = 0.086) and narcolepsy (SMD = −1.02, *p* = 0.111) did not reach statistical significance. These non-significant associations were characterized by exceptionally high heterogeneity (I^2^ = 98.4 and 88.3%, respectively), suggesting that these averages mask substantial underlying variability related to differences in patient phenotyping, disease chronicity, or imaging protocols, rather than indicating a true absence of effect.

### Heterogeneity and bias

**Figure 3:**
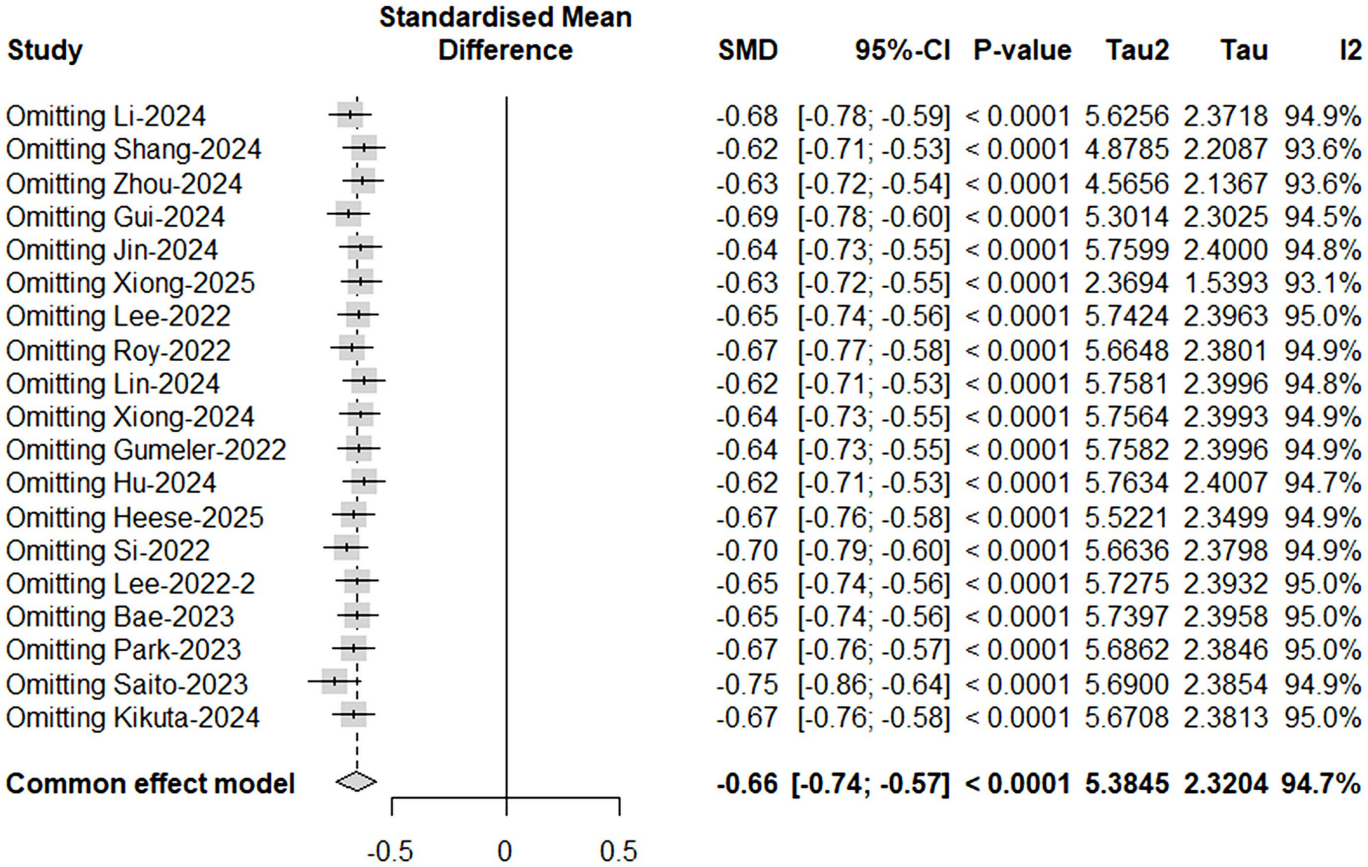
Sensitivity analysis evaluating robustness of pooled results. Sensitivity analyses were performed by sequentially excluding individual studies and recalculating the pooled effect estimates using a fixed-effects model. The consistency of effect sizes across all iterations confirms the robustness of the synthesized outcomes.

Sensitivity analyses, including leave-one-out procedures, confirmed the overall robustness of the primary findings (Figure 3). Pre-specified meta-regression indicated that neither demographic variables (age, gender), sample size, sleep-disorder spectrum, study quality (NOS score), nor MRI parameters (field strength, b value, ROI definition) significantly explained the observed heterogeneity (all *p* > 0.05).

Egger’s regression test indicated substantial publication bias (*p* = 0.010). The adjusted pooled SMD by trim-and-fill analysis remained significant and showed virtually no change (SMD = −1.59, 95% CI [−2.66, −0.54]), indicating that publication bias had little impact on the magnitude of the effect (Supplementary Trim-and-Fill).

To address potential confounding by underlying neurodegeneration, we performed a sensitivity analysis excluding the two studies with comorbid neurodegenerative conditions. The pooled SMD was −1.48 (95% CI [−2.57, −0.39], *p* < 0.001, I^2^ = 93.9%).

## Discussion

This meta-analysis suggests for the first time that glymphatic dysfunction, as assessed by the DTI-ALPS index, may represent a shared neural correlate across a spectrum of sleep pathologies. The substantial pooled effect size (SMD = −1.60) is consistent with major disruption of the brain’s fluid-transport system, positioning glymphatic impairment as a potential pathophysiological amplifier linking disordered sleep to long-term brain health. However, the extreme heterogeneity (I^2^ = 94.7%) substantially limits the interpretability of the global pooled estimate. While this heterogeneity may reflect the etiologic diversity of sleep disorders and their distinct interactions with neurofluid dynamics (29), it also precludes a simple unified interpretation. Therefore, findings should be interpreted primarily through the lens of disorder-specific subgroup analyses.

A key finding is the differential gradient of glymphatic impairment across specific disorders. The most robust deficits were observed in OSA and iRBD—two conditions with well-defined, albeit distinct, mechanisms converging on perivascular clearance. In OSA, chronic intermittent hypoxia is hypothesized to disrupt the polarized expression of astrocytic AQP4 water channels, a cornerstone of glymphatic influx. In iRBD, early accumulation of *α*-synuclein aggregates may physically obstruct perivascular conduits, impeding fluid flow (30, 31). This disorder-specificity suggests that glymphatic dysfunction is not a monolithic outcome but may be mediated by distinct molecular and cellular pathways across different sleep pathologies.

In contrast, the non-significant pooled estimates for insomnia and narcolepsy were accompanied by I^2^ values exceeding 85%, indicating that high heterogeneity prevents firm conclusions; future standardized studies with consistent phenotyping and imaging protocols are needed. The observed variability likely stems from differences in disease chronicity and patient selection rather than indicating a true absence of glymphatic involvement.

A potential confound warranting explicit consideration is the inclusion of studies with comorbid neurodegenerative conditions (Parkinson’s disease, Alzheimer’s disease). Sensitivity analysis excluding these two studies yielded a pooled SMD of −1.48, which remained significant but was attenuated compared to the primary analysis. This suggests that while neurodegeneration contributes to the observed glymphatic impairment, it does not fully account for it. Future studies should carefully disentangle the relative contributions of primary sleep pathology versus underlying neurodegenerative disease.

The inability of meta-regression to identify significant moderators points to the influence of unmeasured technical and biological variables. Notably, DTI-ALPS measurement is highly sensitive to MRI acquisition parameters (e.g., field strength, b-values, ROI definition), which were not uniformly reported. Moreover, DTI-ALPS is an indirect measure; it reflects anisotropic water diffusivity along medullary veins and may be influenced by white matter integrity, small vessel disease, venous architecture, and partial volume effects. It does not directly quantify perivascular fluid transport. Furthermore, reliance on subjective PSQI scores to define “poor sleep” may have introduced phenotypic misclassification, as subjective sleep quality often diverges from objective polysomnographic measures (32). The detected publication bias underscores a literature potentially skewed toward dramatic positive findings, warranting cautious interpretation of the pooled effect magnitude, particularly in smaller subgroups.

Therapeutically, these findings suggest a potential avenue for mechanistically targeted sleep interventions. Preliminary evidence indicating the potential reversibility of this system (33–35) raises the hypothesis that treatments improving sleep architecture or mitigating specific insults (e.g., hypoxia in OSA) could augment waste clearance. However, whether DTI-ALPS can serve as a dynamic biomarker in clinical trials requires prospective validation.

### Limitations and future directions

This study has several limitations inherent to its design. First, the cross-sectional nature of all included data precludes causal inference; we cannot determine whether glymphatic dysfunction precedes or follows sleep disorders. Second, the DTI-ALPS index, while promising, remains an indirect proxy that does not directly measure perivascular fluid transport. It is influenced by microstructural integrity, vascular factors, and technical parameters, which may confound interpretations. Third, the inclusion of studies with comorbid neurodegenerative disease introduces potential confounding; although sensitivity analysis suggested persistence of the effect, residual confounding cannot be excluded. Fourth, the extreme heterogeneity (I^2^ = 94.7%) limits the interpretability of the global pooled effect, and the limited number of studies precluded more comprehensive meta-regression analyses.

Future research should transition to longitudinal designs, integrating DTI-ALPS with molecular neuroimaging (e.g., amyloid-PET), CSF biomarkers, and direct measures of glymphatic function (e.g., perivascular space dynamics) to establish temporal sequence and biological specificity. There is an urgent need for international consortia to standardize DTI-ALPS acquisition and processing pipelines, including consensus on ROI definitions, b-value selection, and quality control procedures. Finally, expanding this research into pre-symptomatic and pediatric cohorts will be vital to determine whether glymphatic dysfunction is a precursor or a consequence of chronic sleep disruption.

## Conclusion

This meta-analysis suggests that glymphatic dysfunction, as assessed by DTI-ALPS, may represent a shared pathophysiological feature across several sleep disorders, particularly OSA and iRBD. However, extreme heterogeneity, publication bias, the inclusion of studies with comorbid neurodegeneration, and the cross-sectional nature of available data warrant cautious interpretation. DTI-ALPS remains an emerging proxy measure; its clinical utility requires validation through longitudinal and interventional studies that directly link it to clinical outcomes and disease progression.

## Data Availability

The original contributions presented in the study are included in the article/Supplementary material, further inquiries can be directed to the corresponding author.
