# Supplementary material for: Glymphatic dysfunction across sleep disorders: a meta-analysis of DTI-ALPS studies
Source: Front Neurol. 2026 Apr 15;17:1789842. doi: 10.3389/fneur.2026.1789842 (PMC13124995; doi:10.3389/fneur.2026.1789842)

Study	Experimental Total	Experimental Mean	Experimental SD	Control Total	Control Mean	Control SD	SMD	95%-CI	Weight (common)	Weight (random)
1	29	1.35	0.0200	32	1.44	0.0100	-5.71	[-6.87; -4.55]	0.6%	5.7%
2	28	1.50	0.1000	21	1.40	0.1000	0.98	[0.38; 1.58]	2.3%	5.9%
3	33	1.39	0.1200	20	1.62	0.1500	-1.72	[-2.37; -1.07]	2.0%	5.9%
4	25	1.38	0.0300	37	1.62	0.0200	-9.67	[-11.50; -7.85]	0.3%	5.2%
5	24	1.30	0.3400	24	1.62	0.2690	-1.00	[-1.60; -0.40]	2.3%	5.9%
6	59	1.34	0.1100	62	1.38	0.1100	-0.36	[-0.72; -0.00]	6.5%	6.0%
7	105	1.70	0.3000	50	2.10	0.4000	-1.19	[-1.55; -0.82]	6.4%	6.0%
8	31	1.27	0.1450	34	1.43	0.1230	-1.20	[-1.73; -0.67]	3.0%	6.0%
9	25	1.70	0.0630	11	1.81	0.0860	-1.59	[-2.40; -0.79]	1.3%	5.8%
10	41	1.44	0.1190	42	1.66	0.1350	-1.69	[-2.19; -1.18]	3.3%	6.0%
11	12	1.45	0.1200	11	1.41	0.1400	0.30	[-0.53; 1.12]	1.2%	5.8%
12	119	1.25	0.1700	129	1.31	0.1700	-0.35	[-0.60; -0.10]	13.4%	6.0%
13	18	1.56	0.2500	18	1.76	0.1990	-0.85	[-1.53; -0.16]	1.8%	5.9%
14	20	1.53	0.1900	20	1.72	0.1900	-0.98	[-1.64; -0.32]	1.9%	5.9%
15	69	1.48	0.2410	51	1.60	0.2450	-0.49	[-0.86; -0.12]	6.2%	6.0%
16	317	1.37	0.0830	515	1.41	0.0820	-0.51	[-0.65; -0.37]	41.8%	6.0%
17	52	1.38	0.1000	52	1.42	0.1000	-0.40	[-0.79; -0.01]	5.6%	6.0%
**Common effect model**	**1007**			**1129**			**-0.65**	**[-0.74; -0.56]**	**100.0%**	**.**
**Random effects model**							**-1.48**	**[-2.57; -0.39]**	**.**	**100.0%**

Heterogeneity: $I^2 = 93.9\%$, $\tau^2 = 5.1488$, $p < 0.0001$

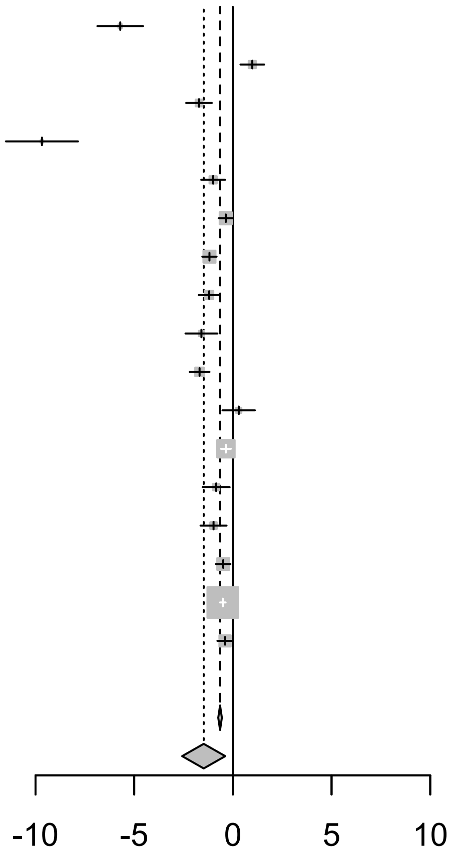

Supplement: Supplementary file 1 [file Data_Sheet_1.PDF]
