# Supplementary figures and images for: Glymphatic dysfunction across sleep disorders: a meta-analysis of DTI-ALPS studies
Source: Front Neurol. 2026 Apr 15;17:1789842. doi: 10.3389/fneur.2026.1789842 (PMC13124995; doi:10.3389/fneur.2026.1789842)

# Trim-and-Fill

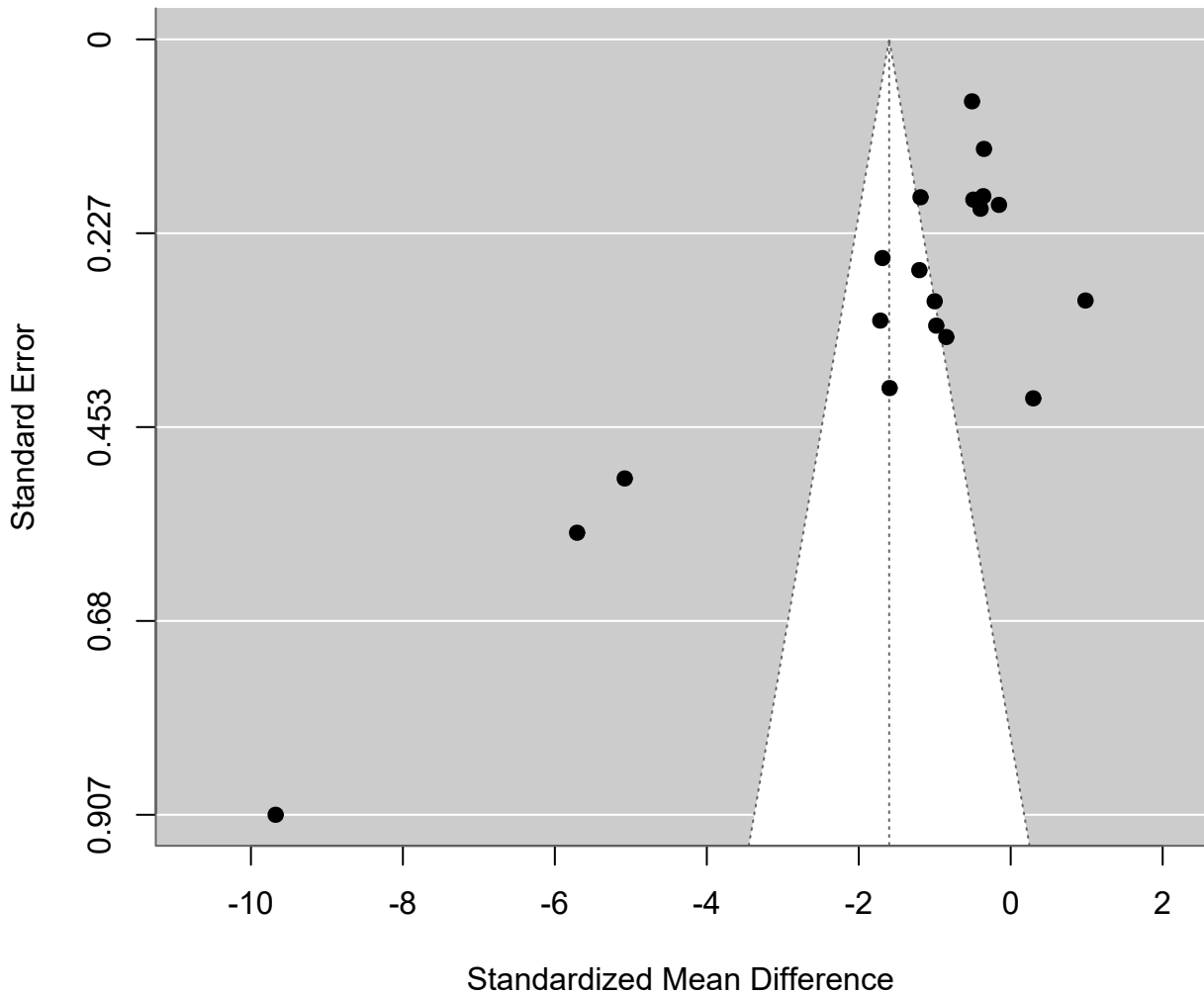

Supplement: Supplementary file 2 [file Data_Sheet_2.PDF]
